# Amyloid Light-Chain (AL) Amyloidosis in a Middle-Aged Lady: Confronting the Challenges of a Rare Diagnosis in a Developing Country

**DOI:** 10.7759/cureus.85493

**Published:** 2025-06-07

**Authors:** Chathurma Piyarathna, Yenifa Samaraweera, Nishadya Ranasinghe, Lakeesha Piyasundara, Mohomed I Mujahieth

**Affiliations:** 1 Department of Haematology, Colombo South Teaching Hospital, Colombo, LKA; 2 Department of Medicine, Colombo South Teaching Hospital, Colombo, LKA

**Keywords:** amyloidosis, chemotherapy, haematological response, monoclonal gammopathy, organ response, proteinuria, transplant

## Abstract

Amyloid light-chain (AL) amyloidosis, which results from monoclonal light-chain deposition from plasma cell dyscrasia, is often identified in old patients. A 37-year-old lady presented with frothy urine and constipation with constitutional symptoms for two months duration. On examination, she had tender hepatomegaly with cervical lymphadenopathy. She was found to have sub-nephrotic-range proteinuria, while serum protein electrophoresis revealed a monoclonal band of 17 g/l. Bone marrow aspiration and biopsy showed evidence of plasma cell dyscrasia with 20% of pleomorphic plasma cells. Myeloma-defining events were negative with normal serum calcium and serum creatinine, a haemoglobin of 10.5 g/dl, no lytic lesions on low-dose whole-body computed tomography, and involved-to-uninvolved serum free light-chain ratio of 8 with involved lambda light chains of 126.21 mg/l (4.23-27.69). Renal and lymph node biopsies demonstrated an eosinophilic amorphous material which showed apple-green birefringence on a Congo red stain suggestive of amyloidosis. Bone marrow aspiration, trephine biopsy, and lymph node biopsy together with renal biopsy led to the diagnosis of AL amyloidosis. She was classified as a stage 1 disease according to the Revised Mayo Clinic staging system. She was treated with a total of nine cycles of cyclophosphamide, bortezomib, and dexamethasone (CycloBorDex), where she achieved a very good partial response as a haematological response and an improvement in 24-hour urine protein excretion as an organ response. This was followed by an autologous bone marrow transplant in the transplant centre. Six months after the bone marrow transplant, it showed a slight progression of disease with an increased difference between involved and uninvolved free light chain (dFLC) with raised 24-hour urinary excretion for which we are planning to restart chemotherapy cycles with VRD (bortezomib-lenalidomide-dexamethasone) as daratumumab is not affordable in our country. Despite the low incidence of AL amyloidosis before the age of 40, this case highlights the monoclonal gammopathy workup in patients with high clinical suspicion, regardless of age. Targeted biopsies should be performed to confirm the diagnosis and start the treatment early to prevent organ failure. Despite the early diagnosis and treatment of AL amyloidosis with conventional chemotherapy, limited accessibility to optimum treatment options in disease progression is a major drawback in managing patients in developing countries.

## Introduction

Immunoglobulin amyloid light-chain (AL) amyloidosis is a clonal plasma cell disorder in which fragments of immunoglobulin light chain or heavy chain are deposited in tissues [1]. According to the global epidemiological data, the majority of AL amyloidosis patients are over 65 years old [2], while few case reports describe amyloidosis at a young age of onset [3]. Even though most of the patients are presented in their seventh decade of life, progression would have happened for several years of its onset [4]. The majority of patients get dominant renal involvement, predominantly a glomerular lesion causing nephrotic syndrome giving rise to generalized oedema [5]. Cardiac involvement usually indicates the advanced stage of disease which has a high risk of death within a few months. However, symptoms are always preceded by detectable monoclonal gammopathy and by elevated biomarkers of organ involvement. Detection of monoclonal gammopathy with early diagnosis and management of amyloidosis will prevent progression into major organ failures. Our patient was diagnosed with AL amyloidosis at 37 years of age with sub-nephrotic-range proteinuria without major organ failures. She was treated with chemotherapy, where she achieved a very good partial response (VGPR) as a haematological response and an improvement in organ response, and then followed by an autologous stem cell transplantation (ASCT). So, with this case scenario, we present the treatment journey of a young amyloid patient, highlighting the importance of evaluating for amyloidosis when clinically indicated, even at young ages. It also emphasizes the obstacles in treating a rare disorder in a resource-poor setting.

## Case presentation

A 37-year-old lady presented with lower abdominal pain and constipation associated with constitutional symptoms such as fatigue and loss of appetite for two months duration. She noticed persistent frothy urine without other urinary tract symptoms. She did not have alopecia, skin rashes, joint pain, fever, haematuria, or generalized oedema.

On general examination, the patient was not pale and anicteric and had no facial puffiness or ankle oedema. However, cervical lymphadenopathy was detected, and the abdominal examination revealed a tender hepatomegaly of 10 cm below the right costal margin. Blood pressure was 130/80 mmHg and pulse rate was 82/min. Clinical examination of cardiovascular, respiratory, nervous, and locomotor systems were within normal limits.

Laboratory investigations (Table 1) revealed a haemoglobin of 10.5 g/dl and a white blood cell count of 9.14×10^9^/l (4-10×10^9^/l), with a platelet count of 172×10^9^/l (150-400×10^9^/l). Other blood reports were as follows: erythrocyte sedimentation rate 130 mm/h, blood urea 2.6 mmol/l (2.8-7.2 mmol/l), creatinine 52 µmol/l (74-110 µmol/l), serum albumin 39 g/l (35-52 g/l), total protein 83.67 g/l (66-83 g/l), total bilirubin 8.46 µmol/l (5-21 µmol/l), alkaline phosphatase 203 U/l (30-120 U/l), alanine transaminase 19 U/l (<50 U/l), aspartate transaminase 32 U/l (<50 U/l), serum sodium 134 mmol/l (136-146 mmol/l), potassium 5 mmol/l (3.5-5.1 mmol/l), and albumin-corrected calcium 2.22 mmol/l (2.06-2.6 mmol/l).

**Table 1:** Baseline investigations at diagnosis ESR: erythrocyte sedimentation rate; ALP: alkaline phosphatase; ALT: alanine transaminase; AST: aspartate transaminase

Investigation	Patient's value	Normal range
Haemoglobin	10.5 g/dl	12-16 g/dl
White blood cells	9.14×10^9^/l	4-10×10^9^/l
Platelet	172×10^9^/l	150-400×10^9^/l
ESR	130 mm/hr	0-20 mm/hr
Blood urea	2.6 mmol/l	2.8-7.2 mmol/l
Creatinine	52 µmol/l	74-110 µmol/l
Serum albumin	39 g/l	35-52 g/l
Total protein	83.67 g/l	66-83 g/l
Total bilirubin	8.46 µmol/l	5-21 µmol/l
ALP	203 U/l	30-120 U/l
ALT	19 U/l	<50 U/l
AST	32 U/l	<50 U/l
Serum sodium	134 mmol/l	136-146 mmol/l
Potassium	5 mmol/l	3.5-5.1 mmol/l
Albumin-corrected calcium	2.22 mmol/l	2.06-2.6 mmol/l
Urine albumin	3+	-
Urine culture	Negative	-

**Figure 1:**
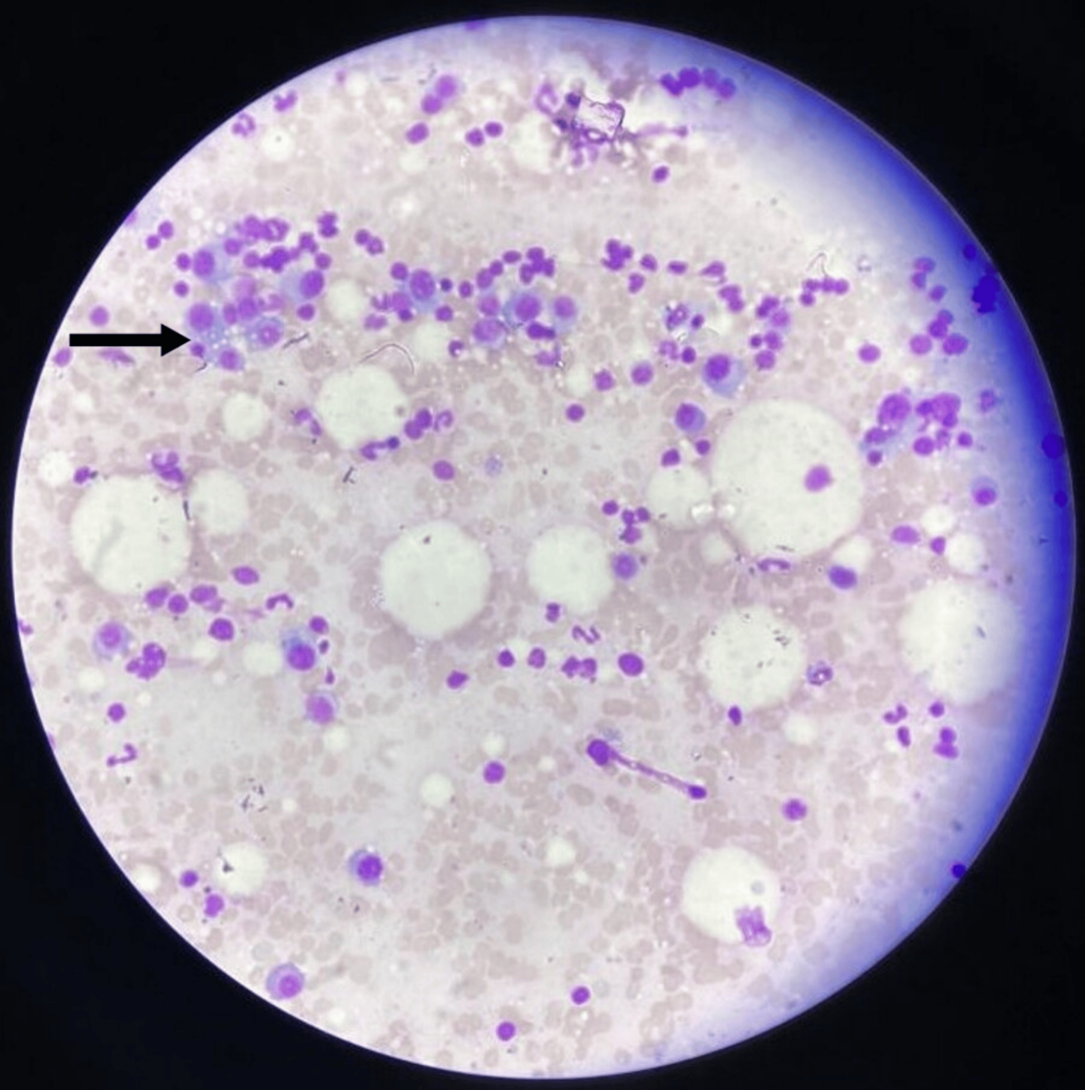
Bone marrow aspirate showing 20% of pleomorphic plasma cells including some bi-nucleated and tri-nucleated forms (Leishman stain: 10×)

**Figure 2:**
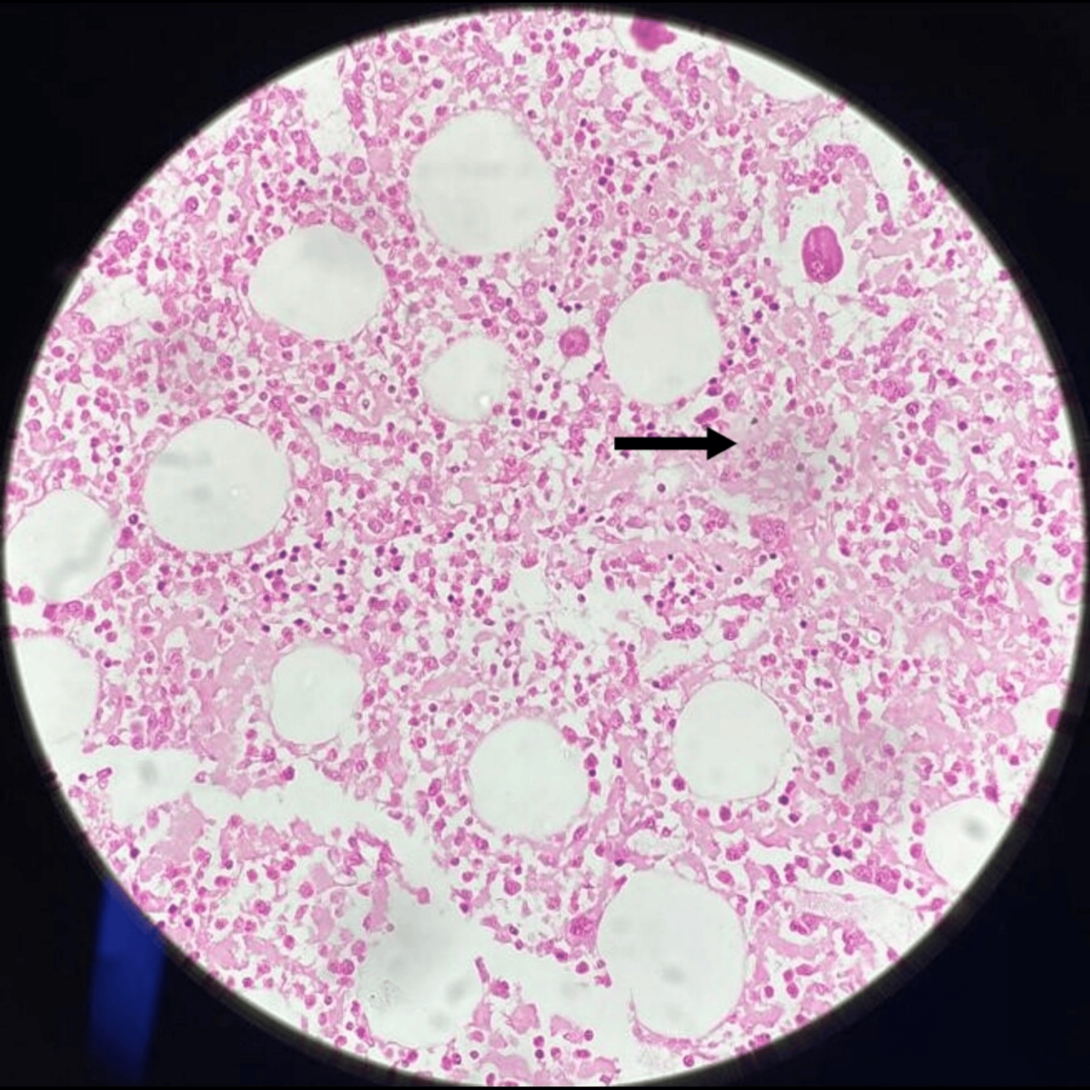
Bone marrow trephine biopsy showing the interstitial deposition of eosinophilic material (H&E stain: 10×) H&E: hematoxylin and eosin

**Figure 3:**
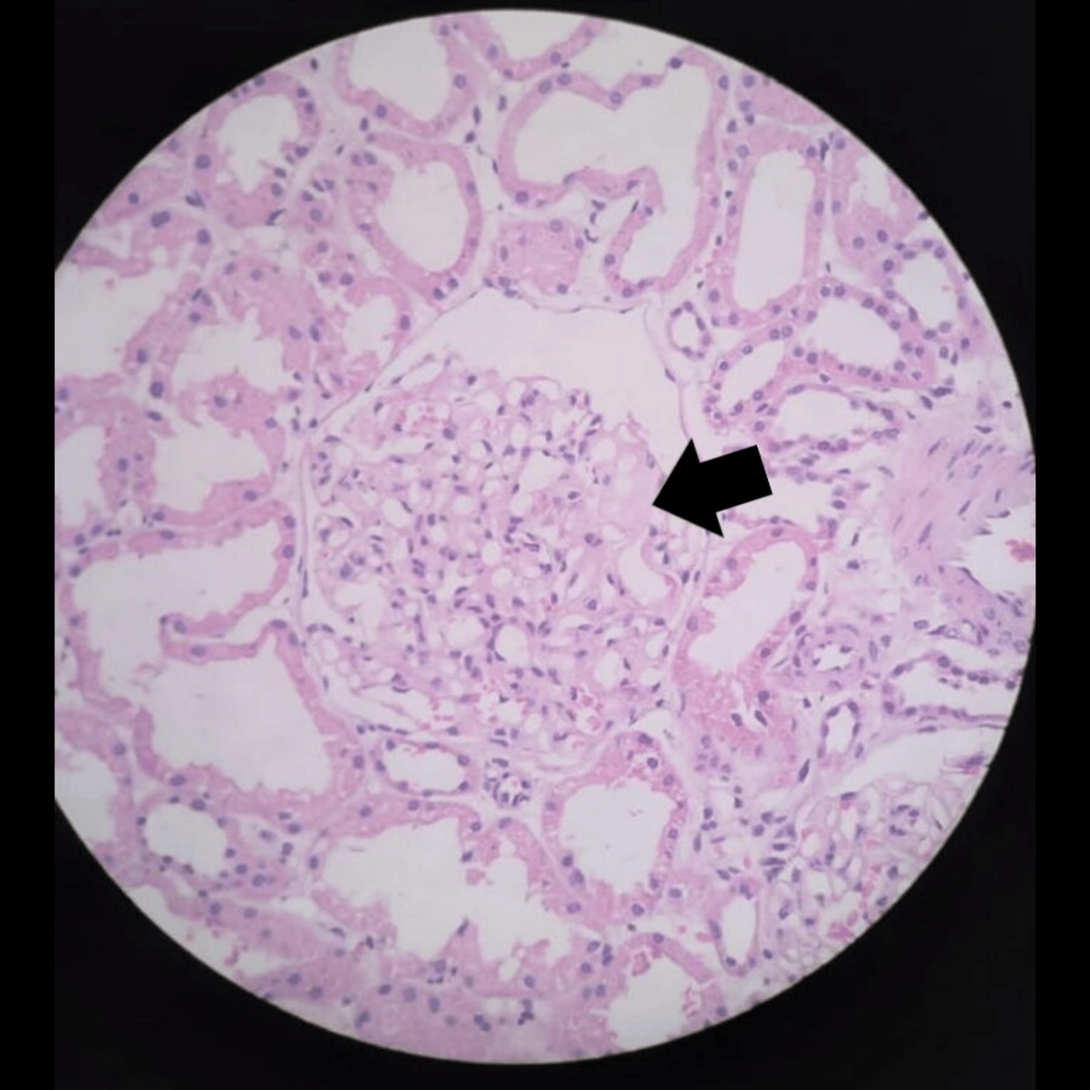
Renal biopsy (H&E stain: 10×). Some glomeruli show variable mesangial matrix expansion by eosinophilic pale material H&E: hematoxylin and eosin

**Figure 4:**
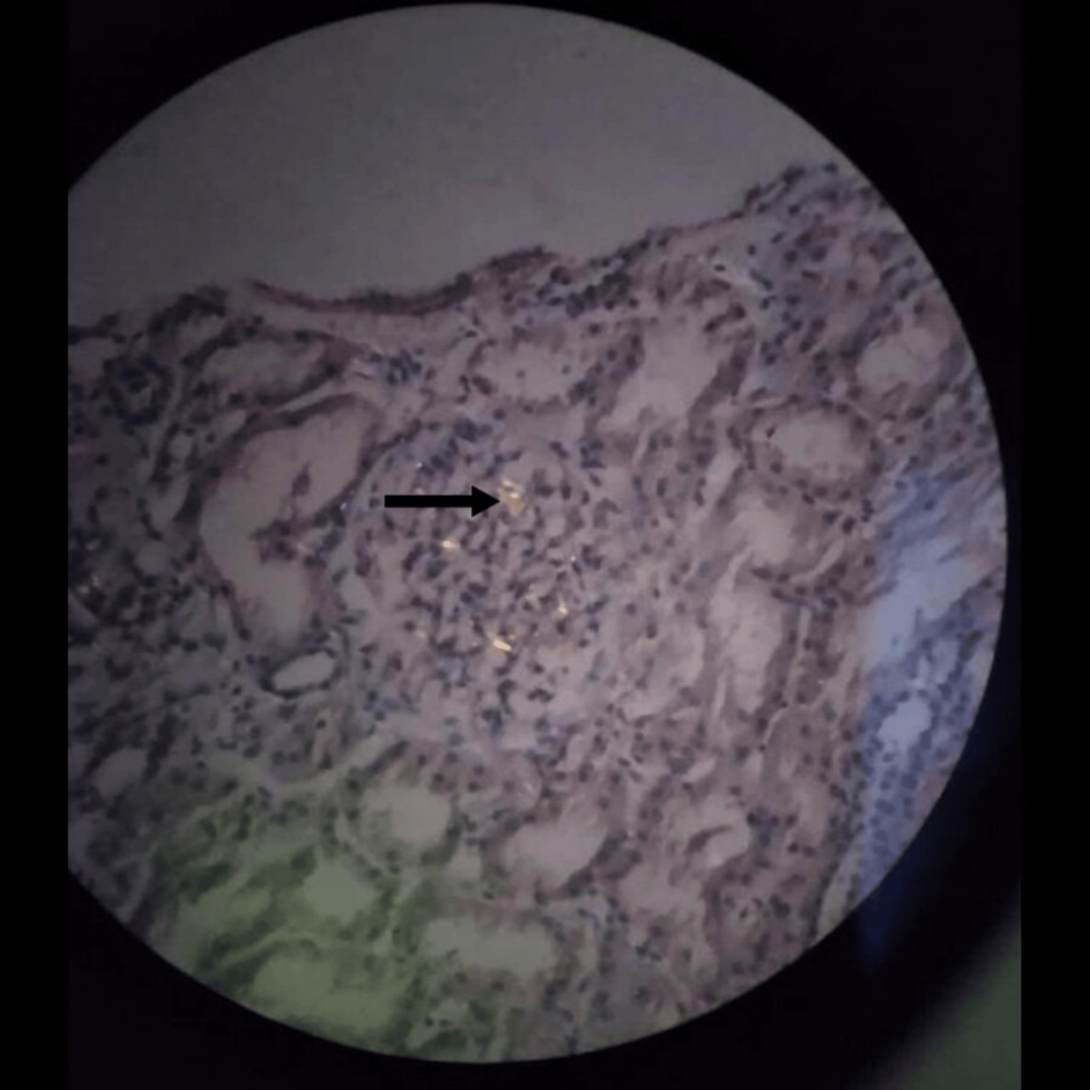
Renal biopsy (Congo red stain). Apple-green birefringence is seen under polarized light around blood vessels and within small amounts of eosinophilic material in the mesangium

**Figure 5:**
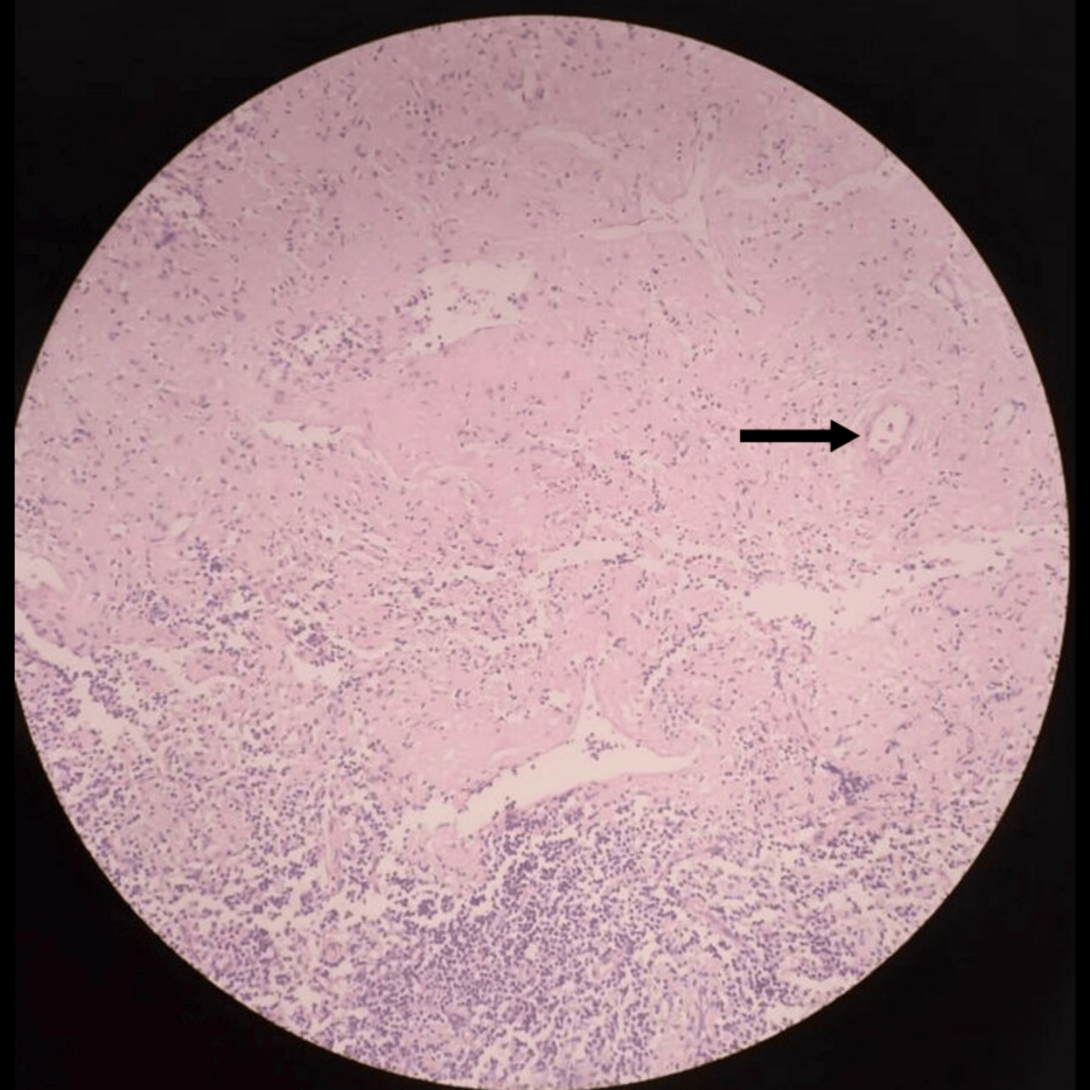
Lymph node biopsy (H&E stain: 10×). The interfollicular region is expanded by homogenous, pale eosinophilic hyaline deposits resembling amyloid H&E: hematoxylin and eosin

**Figure 6:**
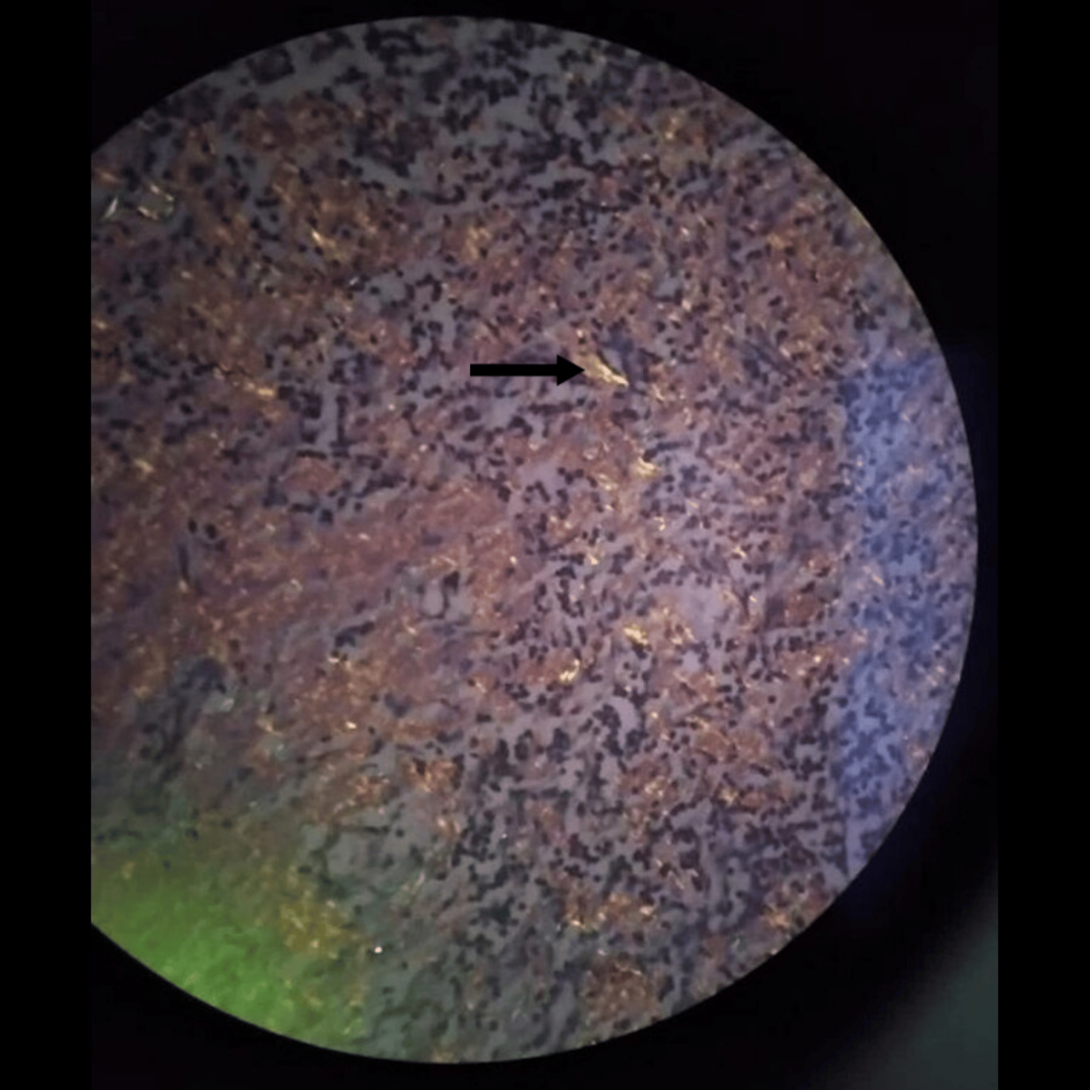
Lymph node biopsy (Congo red stain: 10×). Apple-green birefringence under polarized light predominantly around blood vessels

Urine for microscopic examination revealed albumin 3+. Urine culture was negative. Urine protein creatinine ratio (UPCR) was 1.28 (<0.2), while 24-hour urine protein excretion was 1555 mg indicating sub-nephrotic-range proteinuria. Serum protein electrophoresis showed a monoclonal band in the gamma region with a paraprotein level of 17.7 g/l, and immunofixation revealed the presence of immunoglobulin G (IgG) lambda. The involved-to-uninvolved serum free light-chain ratio was 8 with involved lambda light chains of 126.21 mg/l (4.23-27.69) and kappa light chains of 14.07 mg/l. A monoclonal excretion of heavy-chain and lambda light-chain fragments was noted on urinary immunofixation. The skeletal survey showed no lytic lesion. Bone marrow aspiration and biopsy revealed 20% of pleomorphic plasma cells including some bi-nucleated and tri-nucleated forms (Figure 1). Bone marrow trephine biopsy revealed the interstitial deposition of eosinophilic material (Figure 2) but could not elicit apple-green birefringence on Congo red staining. However, the renal biopsy demonstrated positive apple-green birefringence of Congo red-stained material under polarized light around blood vessels and small amounts of eosinophilic material within the mesangium (Figure 3 and Figure 4). Her abdominal fat pad aspiration was negative for amyloidosis, while lymph node biopsy revealed the amyloid deposits predominantly around blood vessels (Figure 5 and Figure 6).

Even though the proteomic analysis of amyloidotic tissue was not available to identify the protein type, overall results led to the diagnosis of AL amyloidosis. Gastrointestinal (GI) involvement of amyloidosis was excluded by lower GI endoscopy. At the time of diagnosis, she had stage 1 disease according to the Revised Mayo Clinic staging system (troponin T: 0.0037 ng/ml (<0.025 ng/ml), N-terminal pro-B-type natriuretic peptide (NT-proBNP): 40.7 pg/ml (<1800 pg/ml), and difference between involved and uninvolved free light chain (dFLC): 111.9 mg/dl (<180mg/l)).

The pre-treatment evaluation was done with hepatitis B and C, HIV serology, fasting blood sugar, direct antiglobulin test (DAT), and coagulation screening. At the same time, the patient was further evaluated for amyloid-related complications by nerve conduction studies and a 2D echocardiogram which revealed normal findings. After evaluation, she was started on cyclophosphamide, bortezomib, and dexamethasone (CycloBorDex) treatment protocol. The protocol includes bortezomib 1.3 mg/m^2^ administered subcutaneously on days 1, 8, 15, and 22 of the cycle, along with dexamethasone 10 mg orally once daily on the same days as bortezomib. Cyclophosphamide 500 mg is given orally on days 1, 8, 15, 22, and 29 of the cycle, following a weekly dosing schedule. Prophylactic acyclovir, fluconazole, and proton pump inhibitors were continued as concurrent medications. After three cycles of treatment, she achieved a VGPR as a haematological response with dFLC of 16.78 mg/l (<40 mg/l), but disease progression was noticed with organ response as 24-hour urine protein excretion was increased from 1555 mg to 3537 mg per 24 hours. She was started on an angiotensin receptor blocker, irbesartan, while optimizing her glycaemic control to reduce the progression of proteinuria. She was referred to the transplant centre for ASCT. Further six cycles (a total of nine cycles) of chemotherapy were continued to deepen the response and as a bridging to overcome the delay for autologous transplantation. Before transplantation, she maintained a VGPR with a further reduction of dFLC at 3.81 mg/l (lambda light chains: 15.72 mg/l; kappa light chains: 11.91 mg/l) and a paraprotein level of 2.2 g/l, while an improvement in organ response as 24-hour urine protein excretion was reduced to 691.6 mg per 24 hours.

The patient underwent autologous bone marrow transplantation after melphalan conditioning in the transplant unit. Six months after transplantation, her paraprotein level was maintained at 3.8 g/l, while dFLC was 34.3 mg/l (lambda light chains: 83.2 mg/l; kappa light chains: 48.90 mg/l; kappa/lambda ratio: 0.59) which shows a slight progression of involved light chain. Her 24-hour urinary excretion was raised to 1200 mg/day (Table 2). She is currently on follow-up with the plan of restarting chemotherapy cycles with VRD (bortezomib-lenalidomide-dexamethasone) as daratumumab is not affordable in our setup.

**Table 2:** Haematological response and organ response with therapy dFLC: difference in involved and uninvolved free light chain; IgG: immunoglobulin G

	At diagnosis	After 3 cycles of chemotherapy	After 9 cycles of chemotherapy (before transplant)	6 months after transplant
Serum protein electrophoresis (paraprotein level)	17.7 g/l	9.4 g/l	2.2 g/l	3.8 g/l
Serum protein electrophoresis-immunofixation	IgG lambda	IgG lambda	IgG lambda	IgG lambda
Serum free light chains (lambda)	126.21 mg/l	27.12 mg/l	15.72 mg/l	83.2 mg/l
Serum free light chains (kappa)	14.07 mg/l	10.34 mg/l	11.91 mg/l	48.9 mg/l
dFLC	112.14 mg/l	16.78 mg/l	3.81 mg/l	34.3 mg/l
24-hour urine protein excretion	1555 mg	3537 mg	691.6 mg	1200 mg

## Discussion

Amyloidosis is caused by disordered protein folding which causes the deposition of insoluble protein fibrils in extracellular space giving rise to structural and functional tissue disruption [6]. In systemic AL amyloidosis, monoclonal immunoglobulin light-chain fragments are accumulated as beta-pleated sheets most commonly in the kidneys, heart, liver, and peripheral nervous system [5]. It can be related to multiple myeloma or other B-cell lymphoproliferative disorders such as lymphoplasmacytic lymphoma, marginal zone lymphoma, etc. [5].

According to the global epidemiological data, the majority of AL amyloidosis patients are over 65 years old, while the mean age of diagnosis is 63 years [2,7]. Only a few case reports are described about amyloidosis at the young age of onset [3].

Even though most of the patients presented in their seventh decade of life, progression would have happened for several years of its onset. This case underscores the importance of considering amyloidosis in the differential diagnosis when clinical findings are consistent, regardless of the patient's age.

Constitutional symptoms such as fatigue and weight loss can be related to many other causes other than amyloidosis. However, associated proteinuria and clinical findings of hepatomegaly and lymphadenopathy have led to further evaluation with serum protein electrophoresis. The presence of a monoclonal band in serum protein electrophoresis was a key finding that we further investigated with bone marrow, renal, and lymph node biopsy.

Amyloid is a histological diagnosis, by taking a biopsy of an affected organ and staining it with the special stain Congo red [5]. Amyloid deposits produce pathognomonic apple-green birefringence when viewed under cross-polarized light in Congo red-stained tissues. The abdominal fat pad, rectal/labial salivary glands, and bone marrow are the other sites for biopsy. Imaging studies with serum amyloid P component (SAP) scintigraphy would demonstrate the presence and distribution of amyloid deposits in a quantitative manner. Bone uptake is very specific for AL amyloidosis [6]. SAP scintigraphy is not available in our setup.

The type of fibril can be identified by immunohistochemical staining of amyloid-containing tissue sections using a panel of antibodies against the known amyloid protein [6]. Proteomic identification of amyloid type through mass spectrometry, using amyloidotic material cut out from tissue sections by laser dissection, is another method of fibril typing [6]. Sequencing of the genes associated with hereditary amyloidosis should be performed to exclude hereditary amyloidosis [6]. The presence of a paraprotein does not per se confirm a diagnosis of AL amyloidosis which needs the exclusion of serum amyloid A protein (AA), transthyretin amyloidosis (ATTR), and hereditary forms [5]. Despite the less availability of the above investigations, considering the rarity of hereditary forms in the Sri Lankan population, and by excluding other systemic disorders, we assumed AL amyloidosis as the fibril type in our patient.

To evaluate underlying plasma cell dyscrasia, bone marrow aspirate, biopsy with light-chain immunophenotyping/immunohistochemistry, serum and urine electrophoresis, and serum free light-chain assay can be done. If the difference between the involved and uninvolved FLC is ≥50 mg/l at diagnosis, a serum free light-chain assay is used for disease monitoring [5]. Further evaluation with full blood count (FBC), serum creatinine, serum calcium, albumin, skeletal survey, cytogenetics by fluorescence in situ hybridization (FISH), and lymph node biopsy will detect underlying plasma cell dyscrasia. Despite the presence of 20% pleomorphic plasma cells in the bone marrow and an M-protein level of 17 g/l, no myeloma-defining events were identified in our patient.

The majority of patients got dominant renal involvement, predominantly a glomerular lesion causing nephrotic syndrome giving rise to generalized oedema [5]. Our patient had only frothy urine despite having sub-nephrotic-range proteinuria. Altered bowel habits are commonly related to GI involvement as well as autonomic neuropathy where we excluded the GI involvement by endoscopy and biopsy. Hepatomegaly is present in one-quarter of patients, while lymphadenopathy can be a feature of systemic or localized AL amyloidosis [5], where we found both of those features in our patient.

Evaluation of organ involvement should include biopsy of affected organs, liver function tests, serum albumin, renal function tests including serum creatinine, creatinine clearance, and 24-hour urine protein. Additional assessments should include a coagulation screen, NT-proBNP, troponin levels, thyroid function tests, echocardiography, electrocardiogram (ECG), SAP scintigraphy (if available), nerve conduction studies, and cardiac MRI as clinically indicated. In our patient, cardiac and peripheral nervous system involvement was not identified other than renal involvement and liver function derangement.

Organ involvement in amyloidosis determines the clinical manifestations which cause the late recognition of symptoms. Cardiac involvement usually indicates the advanced stage of disease which has a high risk of death within a few months. However, symptoms are always preceded by detectable monoclonal gammopathy and by elevated biomarkers of organ involvement. Detection of monoclonal gammopathy with the early diagnosis and management of amyloidosis will prevent progression into major organ failures [4]. Fortunately, we could diagnose our patient in the early stage of the disease with mild renal impairment without any major organ failures.

Management of AL amyloidosis involves a chemotherapy regimen based on multiple myeloma, with the goal of suppressing the underlying plasma cell dyscrasia. This, in turn, reduces the production of monoclonal light chains, allowing for the gradual regression of amyloid deposits [4]. The goal is rapid and deep haematological response and, if it is not reached, to start on rescue therapy [8]. Organ response can be sometimes delayed as seen in our patient.

Measurement of serum free light chain is the most effective method of monitoring clonal disease. Haematological response in AL amyloidosis is categorized as follows: a partial response is defined by a ≥50% reduction in the dFLC; a VGPR is characterized by a dFLC level of less than 40 mg/l; and a complete response is defined by the normalization of free light chain levels, a normal kappa/lambda ratio, and negative serum and urine immunofixation [8]. Measuring dFLC should be used to monitor haematologic response as long as dFLC is >50 mg/l at diagnosis. The M protein can be used if >5 g/l. Organ response can be assessed by evaluating cardiac, renal, hepatic, and other organ functions, depending on the specific organs involved [8].

In low-risk patients without cardiac involvement and no contraindications to ASCT, bortezomib-based induction therapy should be considered, particularly if bone marrow plasma cell infiltration exceeds 10% or if a delay in ASCT is anticipated, due to its high rates of deep and durable haematologic responses. It can be followed by ASCT after conditioning with high-dose melphalan [4]. Our patient was managed as above.

ASCT provided superior event-free survival (EFS)/overall survival (OS) than chemotherapy alone in retrospective reviews. It remains an important consideration for patients eligible to undergo this technique safely and who fail to achieve ≥VGPR to induction therapy. Renal and cardiac organ responses and high complete haematologic response rates have been reported after ASCT [1].

Progression from partial response is defined as a 50% increase in serum M protein to >5 g/l or a free light-chain increase of 50% to >100 mg/l. There is no standard treatment for relapse. Treatment needs to be tailored to the individual patient and the response to previous treatment regimens. Lenalidomide at a reduced dose is considered in relapsed disease. Even though daratumumab is a highly effective anti-plasma therapy in the treatment of upfront as well as in relapsed disease, it is not affordable in our setup. So currently, the patient is on follow-up with the plan of restarting chemotherapy cycles with VRD for the slow progression of her haematological and organ parameters.

Supportive therapy is an important aspect in the management of AL amyloidosis. It includes the management of renal involvement by loop diuretics, salt restriction, and careful fluid management alongside appropriate anti-hypertensive therapy. Additionally, comprehensive care includes the treatment of congestive cardiac failure, orthostatic hypotension, prevention and treatment of bleeding and thrombotic events, as well as the provision of nutritional and psychosocial support. Multidisciplinary care including haematologist, cardiologist, nephrologist, neurologist, and physician is needed. In our patient, supportive therapy with a multidisciplinary treatment approach was directed to avoid the progression of proteinuria while excluding disease involvement of other organs.

## Conclusions

Amyloidosis is a rare disease that can be complicated due to a long delay in diagnosis causing major organ impairment. Morbidity and mortality can sometimes be limited if diagnosed earlier. Despite the low incidence of AL amyloidosis before the age of 40, monoclonal gammopathy workup is an important aspect in patients with compatible clinical picture, regardless of age. Targeted biopsies should be performed to confirm the diagnosis and start the treatment early to prevent organ failure. Despite the early diagnosis and treatment of AL amyloidosis, limited accessibility to optimum treatment options like daratumumab in disease progression is a major drawback in managing patients in developing countries.
